# Nutritional Therapy in Persons Suffering from Psoriasis

**DOI:** 10.3390/nu14010119

**Published:** 2021-12-28

**Authors:** Jagoda Garbicz, Beata Całyniuk, Michał Górski, Marta Buczkowska, Małgorzata Piecuch, Aleksandra Kulik, Piotr Rozentryt

**Affiliations:** 1 Department of Toxicology and Health Protection, Faculty of Health Sciences in Bytom, Medical University of Silesia in Katowice, 41-902 Bytom, Poland; mbuczkowska@sum.edu.pl (M.B.); piegosia@gmail.com (M.P.); akulik@sum.edu.pl (A.K.); prozentryt@sum.edu.pl (P.R.); 2 Department of Human Nutrition, Faculty of Health Sciences in Bytom, Medical University of Silesia in Katowice, 41-808 Zabrze, Poland; bcalyniuk@sum.edu.pl; 3 Doctoral School of the Medical University of Silesia in Katowice, Faculty of Health Sciences in Bytom, Medical University of Silesia in Katowice, 41-902 Bytom, Poland; mgorski@poczta.onet.eu

**Keywords:** psoriasis, skin, nutrition, diet, fatty acids, obesity

## Abstract

Psoriasis is a chronic inflammatory skin disease. Immunological, genetic, and environmental factors, including diet, play a part in the pathogenesis of psoriasis. Metabolic syndrome or its components are frequent co-morbidities in persons with psoriasis. A change of eating habits can improve the quality of life of patients by relieving skin lesions and by reducing the risk of other diseases. A low-energy diet is recommended for patients with excess body weight. Persons suffering from psoriasis should limit the intake of saturated fatty acids and replace them with polyunsaturated fatty acids from the omega-3 family, which have an anti-inflammatory effect. In diet therapy for persons with psoriasis, the introduction of antioxidants such as vitamin A, vitamin C, vitamin E, carotenoids, flavonoids, and selenium is extremely important. Vitamin D supplementation is also recommended. Some authors suggest that alternative diets have a positive effect on the course of psoriasis. These diets include: a gluten-free diet, a vegetarian diet, and a Mediterranean diet. Diet therapy for patients with psoriasis should also be tailored to pharmacological treatment. For instance, folic acid supplementation is introduced in persons taking methotrexate. The purpose of this paper is to discuss in detail the nutritional recommendations for persons with psoriasis.

## 1. Introduction

Psoriasis is one of the most common inflammatory skin diseases [1]. As estimated by the WHO (World Health Organization), this dermatosis affects 0.09–11.43% of the global population, and the number of patients varies from 1.50% to 5.00% in developed countries [2] It consists of abnormal hyperplasia of keratinocytes (epidermal cells), which leads to the formation of psoriatic plaques [3].

It is a chronic disease in which we can observe periods of spontaneous regression followed by relapses [1]. The disease affects the skin but is also a systemic disease [3].

Immune disorders, which lead to increased pro-inflammatory cytokine production, contribute to the pathogenesis of psoriasis. An increase in the activity of Th1, Th17, and Th22 lymphocytes leads to the production of pro-inflammatory factors in excessive amounts. These factors include: C-reactive protein (CRP), interleukins 1, 2, 6, 8, 12, 17, 22, 23 (IL), interferon γ (IFN-γ), tumour necrosis factor (TNF-α), ceruloplasmin, α2-macroglobulin, α1-antitrypsin, and others. Concentration of these factors is increased both in the acute phase of psoriasis and in remission [1,3,4]. TNF-α plays a key role in the pathogenesis of psoriasis due to its stimulating effect on the proliferation of keratinocytes [5].

Apart from immune disorders, genetic and environmental factors also play a part in the pathogenesis of the disease [1]. Among other things, a relationship between the occurrence of psoriasis and genes in the HLA complex (in particular HLA-Cw6) has been demonstrated. However, it is often the case that the disease never develops in people carrying psoriasis-related genes [1,6].

The environmental factors that can lead to the manifestation of psoriasis or escalation of lesions are as follows [4,6]:

- physical factors (X-rays, subcutaneous and intradermal injections, surgical procedures, vaccinations, tattoos, insect bites, abrasions, burns (including sunburns), acupuncture, UV irradiation);
- chemical factors (chemical burns, topical treatments, others);
- skin diseases (rosacea, fungal infections, allergic contact dermatitis);
- infections (mainly streptococcal pharyngitis, viral infections);
- stress;
- medications (β-adrenolytics, angiotensin-converting enzyme inhibitors, lithium, terbinafine, nonsteroidal anti-inflammatory drugs, anti-malarial drugs, tetracyclines, rapid withdrawal of systemic corticosteroids);
- diet;
- tobacco smoking;
- alcohol consumption.

Despite numerous studies, the etiopathogenesis of psoriasis has not been fully explained [1,7]. It is complex and ambiguous. The above-mentioned factors (immunological, genetic, and environmental) influence the development and severity of this dermatosis to varying degrees. Moreover, it is worth noticing the connection between psoriasis and other diseases [8,9].

Psoriasis is a systemic disease often accompanied by other diseases, e.g., metabolic syndrome and cardiovascular diseases [5,8,10,11]. It is estimated that persons suffering from psoriasis live five years less on average compared to healthy people. The most common causes of death in patients with psoriasis include thromboembolic events and myocardial infarction [10,11].

The chronic inflammatory process is the element that links psoriasis with its co-morbidities [5,7,8,11].

## 2. Metabolic Syndrome

Metabolic syndrome and its components, which include abdominal obesity, atherogenic dyslipidaemia, insulin resistance, impaired glucose tolerance or type 2 diabetes, and hypertension, are observed more frequently in persons with psoriasis than in the general population [8,12,13]. Some people also count the following among the symptoms: hyperhomocysteinaemia, increased concentration of procoagulant factors, microalbuminuria, and non-alcoholic fatty liver disease [1,14,15].

In a population-based cross-sectional study by Langan et al. [16], 34% of persons with psoriasis and 26% of the control group had metabolic syndrome. A positive relationship between the occurrence of metabolic syndrome and the severity of psoriasis (determined by the BSA—Body Surface Area indicator) was also observed. In the studied group, metabolic syndrome was diagnosed in: 32% of patients with mild psoriasis, 36% of those with moderate psoriasis, and as many as 40% of those with a serious form of dermatosis [16]. Thus, the metabolic syndrome is more likely to affect patients with moderate to severe psoriasis, especially patients who developed the disease at a young age [1,16].

Pro-inflammatory cytokines as well as Th1 and Th17 lymphocytes play an important role in psoriasis. Levels of cytokines such as IL-6, TNF-α, angiogenic factors, and adhesion molecules are elevated in obesity psoriasis and ischaemic heart disease. In addition, these inflammatory mediators have been shown to influence fat deposition, insulin action, and lipid metabolism. Thus, chronic inflammation in psoriasis may predispose to diabetes, atherosclerosis, and obesity. On the other hand, inflammatory mediators, whose production accompanies metabolic disorders, may initiate the manifestation of psoriatic lesions or exacerbate existing psoriatic symptoms [10,17].

In patients with psoriasis, TNF-α is found in blood serum and skin lesions, while it is absent in healthy skin. TNF-α is also secreted in adipocytes and has a role in the development of insulin resistance. In addition, the presence of TNF-α leads to an increase in the concentration of free fatty acids and triglycerides in the blood, which may cause atherogenic dyslipidaemia [10,18].

IL-6, found in high concentrations in psoriatic lesions, also plays an important role in metabolic disorders. Its production is three times higher in visceral adipose tissue than in subcutaneous adipose tissue and correlates with the possibility of developing type 2 diabetes. Moreover, elevated IL-6 levels are also found in patients with unstable coronary artery disease [10,18].

## 3. Obesity

Persons suffering from psoriasis are more often overweight or obese compared to the general population [1,19,20].

In the meta-analysis of 18 studies carried out by Armstrong et al. [19], which covered over 200,000 persons suffering from psoriasis, it was calculated that the risk of obesity is over 50% higher in patients with psoriasis compared to those without the disease. In patients with more serious forms of psoriasis, the risk of obesity is higher compared to mild forms of the disease. In addition, patients with normal body weight and psoriasis have a higher risk of becoming obese in the future [19].

Obesity (especially android obesity) promotes the occurrence of psoriasis and worsens its course. On the other hand, psoriasis increases the risk of obesity. Moreover, the more severe the lesions, the higher the risk of obesity is; as BMI increases, the risk of psoriatic arthritis increases [1,6,10,11,14,20]. It has been observed that a body mass index (BMI) > 29 kg/m^2^ is associated with a more than two-fold-increased risk of psoriasis. Furthermore, the severity of psoriatic symptoms is correlated with an increase in BMI [1,20].

BMI is not an ideal indicator for assessing a patient’s nutritional status, as it does not take into account body composition and body fat distribution. Such parameters can be assessed, for example, by using the bioelectrical impedance (BIA) or dual energy X-ray absorptiometry (DXA). In a study by Galluzzo et al. [21], body composition analysis by bioelectrical impedance analysis (BIA) was performed in a group of 164 patients with psoriasis. In that study, 22.50% of men and 5.50% of women with a BMI indicating normal weight and 50% of men and 50% of women with a BMI suggesting overweight were obese according to body fat percentage. This indicates the much greater diagnostic value of the BIA method compared to BMI alone.

Similar results were obtained by Diniz et al. [22]; however, in this study in 42 patients with psoriasis and 41 controls, body weight was measured by DXA. In both study groups, DEXA showed a higher prevalence of obesity compared to BMI and waist circumference.

Blake et al. [23] performed a systematic review of 25 research papers on the relationship between psoriasis and body composition, measured by various methods (BIA, DXA, CT—computed tomography, and others). Their conclusion was that the presence of psoriasis is associated with higher levels of body fat, visceral fat, and reduced muscle mass.

When assessing the nutritional status of patients with psoriasis, it is also worth considering the phase angle measured by BIA. The phase angle allows the assessment of cell size and cell membrane integrity as well as lean body mass and tissue hydration. A decrease in phase angle values suggests cell membrane breakdown and a decrease in intracellular water, while a larger phase angle reflects greater amounts of intact cell membranes and lean body mass. Phase angle may be a predictive marker of mortality in many chronic diseases. Decreased phase angle may be associated with metabolic syndrome and its components [24]. The study by Barrea et al. [24] showed that the phase angle in patients was lower compared to the control group. Phase angle values were correlated with psoriasis patients’ quality of life, disease severity, and the presence of metabolic syndrome.

Studies have shown that when obesity and the HLA-Cw6 gene coexist, the risk of developing psoriasis increases 35-fold compared with the risk in individuals free of these factors [1].

Adipose tissue is the largest endocrine organ, where many proinflammatory cytokines (e.g., IL-6, TNF-α) and bioactive factors called adipokines are produced. These are not only related to metabolic disturbances but may also be responsible for the severity of the psoriatic process [14,15,17,25].

In patients with a severe course of the disease, increased blood levels of pro-inflammatory adipokines (e.g., leptin, visfatin, chemerin) are observed, and during remission their levels decrease. In contrast, anti-inflammatory adipokines (omentin and adiponectin) inhibit the development of psoriatic lesions. Serum levels of these anti-inflammatory factors are significantly lower in patients with severe disease compared to patients with mild forms of psoriasis [1,15].

The higher prevalence of obesity in psoriasis patients may be related not only to the overproduction of pro-inflammatory factors, but also to the fact that psoriasis is often accompanied by stigma and prolonged stress, which often lead to reduced levels of physical activity as well as adverse changes in eating habits and broader lifestyle. These, in turn, may contribute to weight gain [26,27]. 

**Figure 1:**
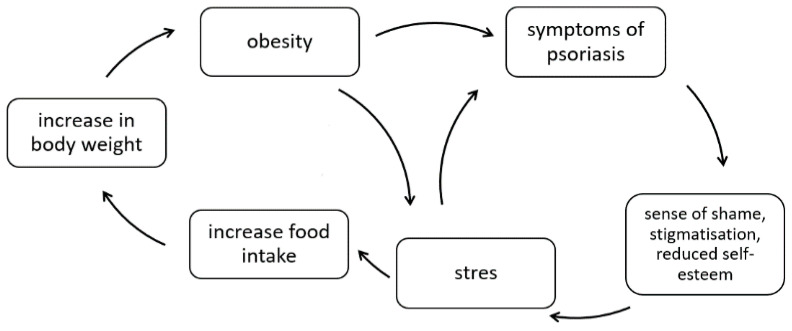
The correlation between psoriasis and obesity in a psychological context (own elaboration based on [26]).

On the other hand, obesity may contribute to lower self-esteem, increased stress levels, and even the development or worsening of depression and anxiety disorders [26]. The relationship between psoriasis and obesity in a psychological context is shown in Figure 1.

**Figure 2:**
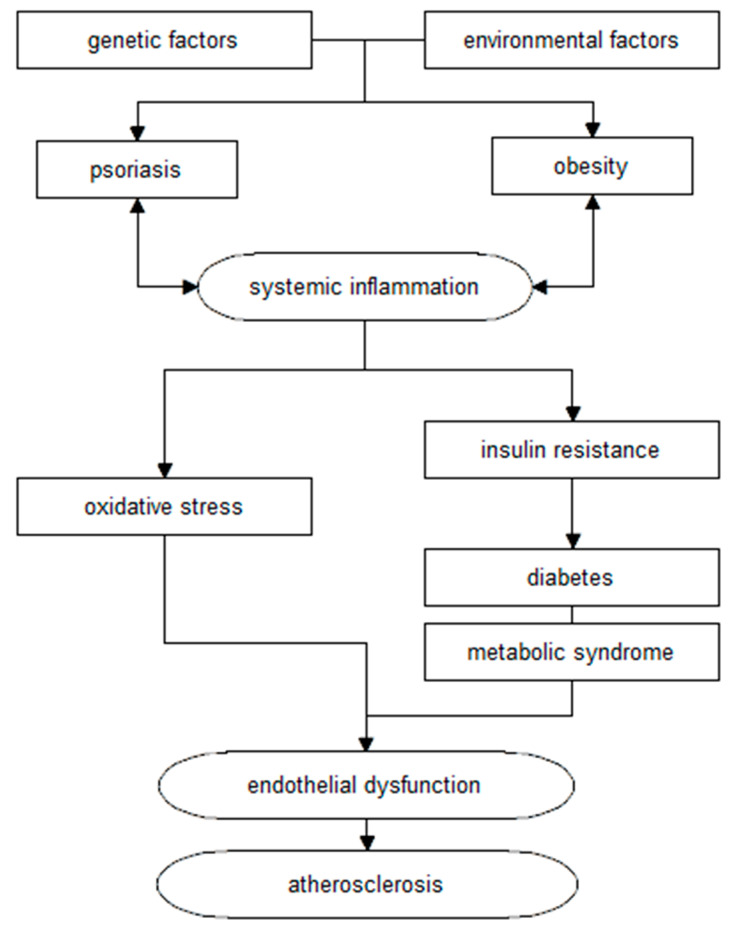
Diagram of the relationship between psoriasis and cardiovascular disease, according to the “psoriasis march” concept (own elaboration based on [1,17]).

Moreover, psoriasis, together with comorbidities (including obesity), leads to a chronic inflammatory process, changes in glucose metabolism, and subsequent development of atherosclerosis and cardiovascular disease. The association of these changes is illustrated by the concept of the so-called “psoriatic march” (Figure 2) [1,17].

To date, no single specific reason for the association between the prevalence of metabolic disorders and psoriasis has been established. However, this relationship has been confirmed by many studies, so every patient with psoriasis should be diagnosed for these conditions, as early implementation of appropriate treatment can prevent the development of these diseases; in addition, dermatologists can play an important role in the early diagnosis and evaluation of metabolic disorders [5,15,28].

Thus, in patients with psoriasis the following is recommended [1,13,15]:

- body weight assessment;
- BMI assessment;
- assessment of waist/hip ratio (WHR);
- fasting blood glucose determination at least once a year;
- more frequent testing for hypertension;
- determination of serum lipids;
- determination of serum uric acid and liver enzymes;
- in patients with other cardiovascular risk factors (e.g., hypertension, obesity), performing an Oral Glucose Tolerance Test (OGTT).

Genetic factors are responsible for the occurrence of psoriasis, yet the manifestation of lesions is determined by environmental factors such as infections, stress, and diet. Therefore, a change of eating habits can significantly improve the quality of life of patients, both through a beneficial effect on psoriatic lesions and through reducing the risk of other diseases, e.g., cardiovascular events [14,29].

A severe course of psoriasis can be associated with nutritional deficiencies caused by faster loss of nutrients, resulting in exfoliation of the affected epidermis [29].

The diet and nutritional status of the patient affect the severity of psoriasis, its course, and the body’s response to pharmacological therapy [11,30].

## 4. Low-Energy Diet in the Treatment of Psoriasis

Obesity maintains systemic inflammation in the body, which can contribute to the intensification of psoriasis symptoms [3]. It is not known, however, whether obesity is a consequence of psoriasis or a risk factor for developing this dermatosis. It is suggested that this relationship is two-way. Obesity is a predisposing factor for psoriasis and intensification of its symptoms, and psoriasis promotes the development of obesity [3,29].

It was observed that a BMI (Body Mass Index) > 29 kg/m^2^ more than doubles the risk of developing this disease, and a reduction in body mass contributes to a reduction of blood serum inflammatory factors, significantly improves the course of the disease, and causes faster regression of psoriatic lesions compared to persons not following the diet [1,14,30].

A randomised study by Jensen et al. [31] proved that a low-energy diet (of 800–1000 kcal/day) followed for a period of up to 8 weeks contributes both to body weight loss (15 kg on average) and to reducing lesions and even improving the Dermatology Life Quality Index (DLQI) [31]. Furthermore, in the following publication, Jansen et al. [32] presented the results of continuing the programme for the next 48 weeks after cessation of the low-energy diet. Although the patients’ body weight increased by 4.90 kg on average compared to the results obtained immediately after implementing a low energy diet, the PASI (Psoriasis Area and Severity Index) was further reduced [32].

An increased risk of side effects is observed in systemically treated obese patients, and body mass reduction leads to a decrease in toxicity of medications and an increase in their effectiveness [29]. A study by Gisondi et al. [33] showed that in obese patients, a 5–10 percent body mass reduction improves the therapeutic response to treatment with cyclosporine A (at a dose of 2.50 mg/kg b.w./day) [33]. Therefore, persons who follow dietary recommendations can reduce the dose of medication, and, consequently, reduce the side effects, including nephrotoxicity [11,30,33]. In turn, in the case of patients after successful therapy with methotrexate, a low-energy diet contributes to prolonged remission of psoriatic symptoms [14]. In persons using biologic medicines and a low-energy diet at the same time, greater relief of psoriatic symptoms was observed (greater improvement in PASI and BSA) compared to the group that underwent biological therapy only, without diet modifications [30].

Thus, in patients with psoriasis, a low-energy diet combined with regular physical activity and possible psychological support focused on patient motivation can complement their therapy [3,4,30,34].

## 5. Selection of Fatty Acids

Selection of the right types of fat plays an important role in the diet of patients with psoriasis. A diet rich in saturated fatty acids found in animal products can increase the risk of cardiovascular diseases. In contrast, consumption of unsaturated fatty acids can contribute to a reduction of the risk of immunometabolic diseases [11].

Monounsaturated fatty acids (MUFA), including oleic acid, protect lipoproteins and cell membranes against harmful oxidative effects. Extra virgin olive oil is a good source of oleic acid [29].

Polyunsaturated fatty acids (PUFA) are not synthesised in the human body so they need to come from food. This group of fatty acids is divided into omega-3 and omega-6 acids. Among fatty acids from the omega-3 family are: α-linolenic acid (ALA), eicosapentaenoic acid (EPA), docosapentaenoic acid (DPA), and docosahexaenoic acid (DHA). The group of omega-6 acids includes linoleic acid (LA) and arachidonic acid (AA) [35].

Polyunsaturated fatty acids are involved in the synthesis of anti-inflammatory or pro-inflammatory compounds. Omega-3 acids have an anti-inflammatory effect, while acids from the omega-6 family have a pro-inflammatory effect [6,14]. For instance, eicosapentaenoic acid competes with arachidonic acid to bind to COX-2 (cyclooxygenase-2) and is a substrate for the synthesis of PGE3 (3-series prostaglandins) and LTB5 (5-series leukotrienes), which have an anti-inflammatory effect. Omega-6 fatty acids have a stimulating effect on the synthesis of pro-inflammatory TNFα, IL-1, and IL-8 [14,34,36,37].

Γ-linolenic acid also belongs to the omega-6 family. However, it is the only one in this group to have an anti-inflammatory effect [14,34].

The diet for patients with psoriasis should be rich in omega-3 fatty acids, while omega-6 acids should be limited [4]. The ratio of fatty acids consumed from the omega-3 and omega-6 acid families should be balanced and amount to 1:1.80 (according to the United States National Institutes of Health Panel) [38]. Other reports suggest that this ratio should be from 1:3 to 1:5 [11,14,35].

A positive relationship between the severity of psoriasis and the ratio of omega-6/omega-3 was noted, and a negative one between PASI as well as EPA and DHA concentration [11].

Patients suffering from psoriasis experience disruption of the unsaturated fatty acid pathway [11,14,34]. Tissue-damaging factors (e.g., topical drugs, UV radiation, bradykinins, histamine, etc.) can stimulate phospholipases in the epidermis. Phospholipase A2 has the ability to release arachidonic acid from cell membranes and initiate its metabolism into pro-inflammatory factors (leukotrienes and prostaglandins). The lipid profile influences changes in eicosanoid and lipid synthesis in psoriatic skin lesions. Increased levels of phospholipase A2 as well as arachidonic acid and its metabolites are found in psoriatic plaques. These factors may increase epidermal cell proliferation and inflammation [39,40].

In addition, omega-3 acids are used to prevent and treat metabolic disorders that often co-occur with psoriasis. Among other things, they have an anti-diabetic effect and also contribute to the improvement of the lipid profile. Moreover, they are a substrate in the process of synthesis of serotonin and dopamine, whereby they are shown to have an antidepressant effect [35].

A low-energy diet (20 kcal/kg of ideal body weight/day) with omega-3 supplementation in patients with psoriasis and obesity improves the metabolic profile and increases the effectiveness of immunomodulatory treatment, which leads to a reduction in PASI and improvement of the quality of life [14].

In the study by Barrea et al. [41], an analysis of fatty acid content in diet was carried out through assessment of seven daily food rations of 41 men with psoriasis compared to a control group. A higher intake of omega-6 acids and lower intake of omega-3 acids was shown in patients with psoriasis compared to the control group. Moreover, an abnormal ratio of omega-6 to omega-3 acids correlated with a higher PASI [41].

Also, in people suffering from psoriasis, after starting a diet with large amounts of marine fish (salmon, mackerel, herring, sardines) an increase in EPA concentration was observed in blood serum along with improvement of psoriatic lesions. Similar effects were shown following the use of fish oils rich in EPA and DHA, corn oil, and also supplementation of omega-3 acid [14,34,35].

In the study by Balbás et al. [28], daily oral supplementation with a preparation containing 640 mg of EPA and DHA was introduced in persons using topical treatment with vitamin D analogues. A quicker improvement in PASI and DLQI (Dermatology Life Quality Index) was observed, compared to persons who only used topical treatments [28]. Similar results were obtained by Adil et al. [37].

In their work, Millsop et al. [36] reviewed studies on omega-3 acid supplementation in patients with psoriasis. In 12 analysed studies, this supplementation was observed to have a beneficial effect, while no significant improvement in the course of the disease was obtained in the 3 remaining ones [36].

Fish are a good source of omega-3 acids: eicosapentaenoic acid (EPA), docosahexaenoic acid (DHA), and docosapentaenoic acid. They should be included in the diet of patients with psoriasis because of their beneficial anti-inflammatory, immunomodulatory, and antioxidant effects [42,43,44]. The validity of fish oil supplementation in patients with psoriasis was not confirmed in a 2019 meta-analysis of 13 randomised controlled trials by Yang et al. [45], while in 2020 a systematic review of 18 randomised controlled trials by Chen et al. [42] confirmed the efficacy of fish oil use in combination with conventional psoriasis treatment.

It is recommended that people with psoriasis consume products rich in omega-3 fatty acids. The daily amount of these fatty acids should be about 1–2 g. Such products include: fatty marine fish (herring, sardines, salmon, tuna, mackerel), seafood, vegetable oils (rapeseed oil, linseed oil, walnut oil), and nuts (mainly walnuts). In contrast, animal fats (saturated fatty acids) and industrial (trans) fats, present, e.g., in stick margarine, highly processed foods, and confectionery products, should be avoided [6,14,34,35].

The introduction of supplements from the omega-3 acid family is an alternative here. It is important to remember that these preparations should be taken with a meal in order to increase absorption in the intestinal mucosa [35].

## 6. Selection of Carbohydrate Products

Excessive consumption of simple sugars (glucose, fructose, sucrose), has been reported to contribute to the exacerbation of psoriasis symptoms [9]. Moreover, the combination of simple sugars and omega-3 acids may reduce the health-promoting effects of these types of fats [35].

A high intake of simple carbohydrates may cause intensification of oxidative stress and exacerbate the course of psoriasis [4,9,29]. 

Dietary fibre has intestinal and systemic anti-inflammatory effects, has a beneficial effect on intestinal microflora, and also contributes to weight loss, as a diet rich in fibre has a lower energy density [9].

It is recommended to choose food products with a low glycaemic index or load (whole grain cereals, unprocessed vegetables and selected fruits), mainly due to the more frequent occurrence of metabolic diseases (such as diabetes, insulin resistance) in patients with psoriasis [9]. Carbohydrate products with a high glycaemic index (e.g., refined sugar, sweets, honey, sweet drinks, fruit preparations, some fruits, white bread, plain pasta, white rice, potatoes) should be restricted in the diet of patients with psoriasis [9,14].

Persons suffering from psoriasis should increase dietary fibre intake, which can help reduce oxidative stress [29]. Fibre is contained in plant products such as vegetables, fruit, and whole grain cereals [9].

## 7. The Importance of Antioxidants

The chronic inflammation that is related to psoriatic lesions has an effect on the formation of free radicals and superoxide anion, resulting in oxidative stress. This term describes an imbalance between the number of reactive oxygen species and antioxidants at the cellular level [11].

Oxidative stress can contribute to atherosclerotic plaque formation. Reactive oxygen species cause damage to the vascular endothelial cells, which leads to an increase in the permeability of small vessels and, in consequence, allows the transmission of inflammatory cells, which in turn intensifies the development of inflammation in psoriasis [11,34].

Antioxidants (flavonoids, vitamin A, vitamin C, vitamin E, β-carotene) are substances that protect against the harmful effects of free radicals through many chemical transformations [14,34,46].

Reports have shown that a diet rich in vitamin C, β-carotene, and flavonoids, which includes green vegetables, carrots, tomatoes, and fruit, helps improve skin lesions [6,34]. Therefore, persons with psoriasis should increase the intake of fresh fruit and vegetables as well as include polyphenol-rich products such as tea, coffee, herbs, and spices in their diet [47].

EPA and DHA acids also contribute to the reduction of oxidative stress [14].

In addition, selenium deficiency, often observed in patients, can be a risk factor for the development of psoriasis, and supplementation of this element suppresses secretion of TNF-α. However, no improvement in psoriasis symptoms was demonstrated in patients taking selenium supplements [14,34,36].

Therefore, patients with psoriasis are recommended to eat products that provide large amounts of antioxidants, i.e., mainly vegetables (Table 1).

## 8. The Importance of Vitamin D_3_

Currently, there is a widespread vitamin D deficiency in European countries. However, patients with severe psoriasis have significantly lower concentrations of 1.25-(OH)_2_D_3_ (calcitriol—the active form of vitamin D) in blood serum than healthy persons or patients with moderate to mild disease [3,30,34]. As estimated, vitamin D deficiencies affect 50% of patients with psoriasis in summer and as many as 80% in winter [51]. Moreover, it has been shown that in the winter months, when skin synthesis of vitamin D is significantly reduced, symptoms often intensify in patients with psoriasis [52].

In a pilot study by Finamor et al. [53], an improvement in PASI was shown in all studied persons with psoriasis after six months of high-dose vitamin D therapy (35,000 IU/day).

In the publication by Gaal et al. [54], it was observed that vitamin D supplementation (0.25 μg 2 times a day, for 6 months) among patients with psoriatic dermatitis has a positive effect on the regulation of immune response.

A paper by Tajjour et al. [55] demonstrated a lower vitamin D concentration in the blood serum of persons with psoriasis compared to the control group. Moreover, the authors of this paper observed a negative correlation between vitamin D concentration in blood serum and PASI. 

The underlying cause of psoriasis is a disturbance of the immune system, which is why it is so important to maintain adequate levels of vitamin D, which has a modulating effect on the cells of the immune system [36,51]. Moreover, vitamin D also contributes to the process of normal growth and differentiation of keratinocytes [14,36,51]. Deficiencies of this vitamin may also increase the risk of metabolic syndrome [56].

Vitamin D has an inhibitory effect on keratinocyte proliferation; decreases psoriasin (a skin peptide, S100A7, whose levels are elevated in the skin of people with psoriasis); increases the synthesis of keratin, tranglutaminase, involucrin, loricrin, and filaggrin in the skin. Vitamin D_3_ increases the synthesis of ceramide, which, by feedback loop, enhances the pro-differentiating effect of keratinocytes by calcitriol. In addition, vitamin D_3_ is involved in regulating the synthesis of glycosylceramides, which are essential for maintaining the integrity of the skin barrier and permeability in the stratum corneum [57].

The authors also suggest that vitamin D deficiencies in psoriasis may be associated with concomitant cardiovascular diseases and obesity [57]. Thus, supplementation of this vitamin can both reduce symptoms caused by psoriasis and contribute to the levelling of the general condition of the patient and the symptoms of other diseases [3,34].

There are few dietary sources of vitamin D_3_. These are mainly animal products (oily fish such as herring, salmon, mackerel, fish oils, and egg yolks). Products fortified with this vitamin are also available (e.g., milk, cereals, juices) [57]. Vitamin D is supplied to the human body mainly through cutaneous synthesis under the influence of ultraviolet B (UV-B) rays [58,59]. When the intake of vitamin D from food and skin synthesis is insufficient, supplementation is recommended [57,58].

Studies have shown that supplementation with 1000 IU of vitamin D_3_ per day leads to an increase in 25(OH)D levels to about 10–20 ng/mL [57]. The European Food and Safety Authority (EFSA) has defined the tolerable upper level intake (UL) of vitamin D for adults as 4000 IU per day [59]. Supplementation with this vitamin should be preceded by determination of serum 25(OH)D concentration [58]. These tests should be repeated after 2–3 months to assess the effectiveness of supplementation and adjust the dose if necessary [57].

There is a lack of large population-based sample studies to determine the dose of vitamin D supplementation in patients with psoriasis [57].

However, too high doses of vitamin D should not be used due to the risk of hypercalcaemia or hypercalciuria [21]. For the same reason, supplementation with this vitamin is not practised in patients treated topically with vitamin D analogues [34].

## 9. Use of Seaweed in Psoriasis

The human microbiota is an environment rich in microorganisms that influences protein, carbohydrate, and lipid metabolism, immune system development, and body homeostasis. The largest microbiota resource in the human body is the large intestine. In the literature, many works show a link between intestinal dysbiosis and the development of diseases with an extraintestinal location, such as multiple sclerosis, type 1 diabetes, systemic lupus erythematosus, or psoriasis [60,61,62,63,64,65,66]. Significant differences have been observed between the gut microbiota of patients with psoriasis and that of the healthy population, suggesting a potential influence of gut dysbiosis on the development of psoriasis [66]. 

Many reports indicate a link between diet modulating microbiota composition and immunostimulatory effects [60,67]. Probiotic bacteria are naturally found in fermented dairy products such as kefir and yoghurt, as well as in pickled vegetables [67,68].

Recent reports indicate a beneficial effect of probiotic supplementation in patients with psoriasis [69,70] Polysaccharides, which are components of dietary fibre, show prebiotic effects, beneficially altering the intestinal microflora. Studies by Takahashi et al. [60] and other reports [71,72] indicate that fucoidan, a sulfated polysaccharide found in the cell walls of brown seaweed, exhibits anticoagulant, anticancer, immunomodulatory, and apoptosis-inducing effects. Fucoidan has also been found to have a positive effect on the intestinal barrier and the composition of the bacterial flora, as well as improving the course of psoriasis [60].

In addition, marine seaweed are a sustainable source of bioactive lipids with high concentrations of omega-3 fatty acids [73,74,75,76] and vitamin D_3_ [77,78], whose beneficial effects in psoriasis patients have been outlined above.

The application of seaweed directly to the patient’s skin also has a beneficial effect on the course of dermatosis. In a study by Grether-Beck et al. [79], it was shown that Blue Lagoon seaweed extract has a biological effect on the skin, influences the expression levels of mRNAs that are relevant for melanin synthesis, and reduces unevenness in skin pigmentation.

## 10. Coffee and Psoriasis

Coffee is one of the most consumed liquids, regardless of geographical region. According to the statistics, only water and tea are more frequently consumed liquids [80]. Importantly, coffee is a pharmacologically active fluid. There are many biologically active substances in its composition [81]. These include carbohydrates, lipids, nitrogenous compounds, minerals, vitamins, phenolic compounds, lactones, diterpenes, antioxidants, alkaloids, and caffeine, which constitutes about 1% of the total composition of coffee [82,83]. This substance exhibits a number of therapeutic actions. It has been demonstrated that it reduces the migration of monocytes and neutrophils, reduces blood glucose levels, has an anti-inflammatory and immunosuppressive effect, and protects against neurodegeneration [81,83,84]. 

The best-studied component of coffee is caffeine. Its action involves inhibition of Th1/Th2 cell proliferation, inhibition of the release of pro-inflammatory cytokines (IL-1, IL-6, IL-11, TNF-alpha), and concomitant release of anti-inflammatory markers such as IL-4, IL-10, and adiponectin [83,85]. Furthermore, it inhibits cyclin adenosine monophosphate (cAMP) phosphodiesterase, which acts as an immunomodulator, stimulates the release of anti-inflammatory cytokines and acts as an adenosine receptor antagonist [82]. 

Hall et al. [86] argue that the anti-inflammatory effects of coffee are related to the presence of substances called polyphenols in its composition. A group of these compounds, especially chlorogenic acid and its metabolites, show strong inhibitory effects on pro-inflammatory cytokines, while caffeic acid reduces nitrite levels and inhibits inflammatory scapes [86]. 

The arabinogalactan proteins present in coffee have an immunosuppressive effect, stimulating splenocytes and peritoneal macrophages, resulting in a reduction in skin inflammation and reducing the severity of allergic reactions [82]. 

Research on the use of coffee in the treatment or support of psoriasis is inconclusive. Zampelas et al. [87] hypothesised that coffee consumption increases the body’s inflammatory process, which may negatively correlate with psoriasis severity. A study conducted by this research team showed that regular coffee consumption increased IL-6, CRP, and TNF-alpha levels, which translated into clinical severity of psoriasis symptoms. However, it is important to note that this study correlated high coffee consumption (>200 mg/day) with psoriasis severity. On the contrary, Li et al. [88] showed that coffee improved the efficacy of pharmacological treatment of psoriasis, especially when methotrexate or sulfasalazine was used. Interestingly, decaffeinated coffee had no effect on the risk of developing psoriasis [88]. The same study also assessed the correlation between caffeine intake and the risk of developing psoriasis. It was shown that the risk of psoriasis moderately correlated with an increase in caffeine intake, although this fact was not statistically significant in cigarette smokers. It should be mentioned here that this study had a very important limitation—the effects of caffeine from many different sources, including sweetened drinks and highly processed foods, were assessed—which may have played a role in the conclusions drawn. Studies by Sharif et al. [85] and Hall et al. [86] have shown that regular coffee consumption increases the concentration of anti-inflammatory factors and reduces the production of pro-inflammatory factors (especially TNF-alpha), which is important in reducing the severity of psoriasis. 

Recent studies [80,89] demonstrate that the effect of coffee is dose-dependent. Regular moderate coffee consumption (up to 3 cups per day) alleviates psoriasis symptoms and has an anti-inflammatory effect, whereas higher coffee consumption (especially >4 cups of coffee per day) exacerbates clinical symptoms of psoriasis, which is associated with an increase in pro-inflammatory substances.

## 11. Alternative Diets in the Treatment of Psoriasis

### 11.1. Gluten-Free Diet

Asymptomatic coeliac disease is observed more frequently in patients with psoriasis compared to the general population [6,14,90]. A meta-analysis on the association between psoriasis and celiac disease concluded that patients with this dermatosis have an approximately three-fold increased risk of celiac disease [91]. Meanwhile, the risk of psoriasis in patients with coeliac disease is higher than in general population [11].

In coeliac disease, as a result of eating gluten-containing cereals (wheat, rye, barley, oats, triticale), inflammation of the small intestine mucosa and intestinal villous atrophy occurs, which in turn leads to absorption disorders [34].

Interestingly, coeliac disease, like psoriasis, belongs to the group of autoimmune diseases in which the body attacks its own tissues mistakenly using the immune system [92].

In order to diagnose coeliac disease, the antibodies to tissue transglutaminase, gliadin, and smooth muscle endomysium are measured. It is worth noting that patients with psoriasis have the tissue transglutaminase and gliadin antibodies more often compared to the control group [14,92,93]. A relationship has also been observed between the occurrence of these antibodies and intensification of psoriasis lesions [14].

It has not been clearly established whether gluten consumption can contribute to the development of psoriasis [90,94].

Reports confirm improvement of psoriasis lesions in patients who eliminated gluten from their diet [90,91,95]. This applies both to persons with concomitant coeliac disease and patients without coeliac disease but with gliadin antibodies present. Moreover, after returning to a traditional diet containing gluten, a deterioration of psoriatic lesions was observed [11,14,34]. However, in patients without antibodies present, no improvement in lesions was noted [14].

Starting a gluten-free diet in patients with psoriasis may be controversial, and further research is needed to explain the role of this nutritional plan in detail [92,93].

### 11.2. Vegetarian Diet

Some authors draw attention to the positive impact of a vegetarian diet on the course of the disease in patients with psoriasis. The diet consists of giving up eating meat products while increasing the intake of vegetables, fruits, legumes, nuts, and cereal products. As a result, the diet is low in saturated, trans, and arachidonic acid as well as high in antioxidants and omega-3 fatty acids [11,14].

Moreover, a vegetarian diet may contribute to the levelling of potassium deficiencies. An increase in potassium intake may cause an increase in the synthesis of cortisol, which has an anti-inflammatory effect. It is known that a diet rich in vegetables and fruit may significantly contribute to improving the clinical condition of persons suffering from psoriasis [14].

### 11.3. Mediterranean Diet

The Mediterranean diet is characterised by a high consumption of vegetables, cereals, legumes, fish, fruit, and nuts. Extra virgin olive is the main source of fat in this diet. In this diet, it is recommended to consume small amounts of wine with meals [29,46].

The consumption of meat, dairy products, and eggs is limited. Animal fats such as butter, cream, and lard are not included in this diet [29,46].

It is suggested that the Mediterranean diet is associated with prevention of metabolic, cardiovascular, and chronic inflammatory diseases [29,46].

The health-promoting properties of this diet are linked with high consumption of [29,46]:

- products with antioxidant and anti-inflammatory effects (fruit, vegetables, red wine, natural herbs);
- unsaturated fatty acids (fish, olive oil, nuts);
- products that are a source of dietary fibre;
- products that are a source of vitamins and minerals;
- and low consumption of:
- products that are sources of saturated fat;
- simple carbohydrates;
- highly processed products.

In the study by Barrea et al. [46], the relationship between adherence to a Mediterranean diet and severity of psoriasis was assessed. PASI was negatively correlated both with the level of adherence to the diet and higher consumption of extra virgin olive oil, vegetables, fruits, legumes, fish, and nuts, and positively correlated with higher consumption of red meat. Furthermore, patients with psoriasis followed a Mediterranean diet to a lower extent compared to the control group [46].

The publications of Korovesi et al. [96], Phan et al. [97], Molina-Leyva et al. [98], and Caso et al. [99] also confirm the inverse relationship between the use of the Mediterranean diet in patients and the severity of psoriasis or psoriatic arthritis.

### 11.4. Ketogenic Diet

Regardless of adipocytokine levels, body fat, body weight, and glycemic values, saturated fatty acid intake is a significant exacerbator of psoriasis [100,101].

A ketogenic diet based on increased fat intake (75–80% kcal from fat, 5–10% kcal from carbohydrates, and 15–25% kcal from protein) increases ketone bodies, may have anti-inflammatory effects, and lowers blood glucose levels [102]. The high content of medium-chain triglycerides (MCT) with a stronger anti-inflammatory effect compared to long-chain triglycerides (LCT), as well as the anti-angiogenic nature and the high amount of omega-3 fatty acids, make the ketogenic diet a potentially beneficial nutritional intervention in patients with psoriasis [102].

Excess body weight and systemic pro-inflammatory activation are important risk factors for the development of psoriasis. Recent reports [103,104,105,106] indicate that a low-calorie ketogenic diet (VLCKD) (which leads to both weight loss and a reduction in chronic inflammation) can reduce the severity of clinical symptoms and even inhibit psoriatic disease triggering. This nutritional regimen may be a potential first-line therapy for patients with psoriasis and obesity.

The VLCKD medical protocol is a nutritional programme based on high-biological-value protein and natural foods [104]. The scheme consists of three phases: active, re-education, and maintenance. The active phase involves a very low calorie diet (600–800 kcal/day), low intake of carbohydrates (<50 g per day from vegetables) and lipids (only 10 g of olive oil per day), and a normal supply of high-biological-value protein (0.80–1.20 g per kilogram of appropriate body weight), which maintains lean body mass and covers the body’s daily protein requirements. The re-education phase consists of gradually introducing more products into the diet and increasing the caloric intake (800–1500 kcal/day), as well as educating patients in order to maintain body weight in the long term. The maintenance phase, which is the final stage, consists of a balanced diet (1500–2000 kcal/day) [104].

It seems reasonable to undertake further studies on the application of the VLCKD protocol in patients with psoriasis and to compare the results of these studies with the effects of other dietary schemes.

## 12. Diet Therapy and the Use of Medicines

Nutrition of patients with psoriasis should also be tailored to their therapy.

The use of methotrexate contributes to an increase in the concentration of toxic homocysteine and reduces the blood level of folic acid. Moreover, deficiencies of this vitamin are also associated with increased homocysteine levels. Supplementation with folic acid, usually in the amount of 10–15 mg/week, should be introduced in patients taking methotrexate. Supplementation regimes vary, but the vitamins should always be administered at least 12 or 24 h (according to various reports) after taking methotrexate due to the risk of a decrease in the effectiveness of the drug [30,107]. This is possible because methotrexate is taken once a week [107]. It should be remembered that the bioavailability of folic acid decreases when it is taken with a meal [107]. Supplementation of this vitamin in persons taking methotrexate can also contribute to relief of other adverse reactions related to the bone marrow, gastrointestinal tract, and liver, caused by the medication [107].

It should also be noted that methotrexate may cause nausea, which in turn is a common cause of loss of appetite in patients and may contribute to the development of deficiencies of certain nutrients. Therefore, it is necessary to include a balanced diet, providing all the necessary components in the proper amounts [30].

Cyclosporin A may increase the risk of hypertension. In one study, patients starting therapy with this drug adhered to a low-sodium diet, followed by a high-sodium diet. After four months of treatment with a low-sodium diet, blood pressure did not increase, whereas in the next period, when patients adhered to a high-sodium diet, there was a significant increase in both systolic and diastolic pressure [30].

Administration of cyclosporin with grapefruit juice results in an increase in bioavailability of the drug by up to 60%. Therefore, patients taking this substance should avoid grapefruit juice, grapefruit, and other citrus fruits [6,14,30].

Vitamin A derivatives are also used in psoriasis. It should be remembered that this may result in hypervitaminosis, especially in patients who decide to use additional supplementation of this vitamin and take large amounts of products rich in vitamin A. Retinoids can also cause increased cholesterol and triglyceride levels in blood serum. Therefore, it is recommended to eat products rich in polyunsaturated fatty acids from the omega-3 family, and to limit the consumption of simple sugars and alcohol [14].

## 13. Alcohol and Intensification of Psoriatic Lesions

Studies have shown that patients with psoriasis tend to consume alcohol in excess more often. However, it cannot be clearly determined whether the occurrence of psoriasis correlates with the occurrence of alcohol addiction [1,6,14,94]. In addition, it is not known whether the decreased quality of life of patients with psoriasis results in excessive alcohol consumption, or whether alcohol consumption provokes psoriatic symptoms [1,5].

However, it was noted that alcohol consumption may contribute to the intensification of psoriatic lesions [6,14,94].

In the study by Qureshi et al. [94], covering over 100,000 American women, increased risk of psoriasis was observed in the case of higher alcohol consumption. The risk of psoriasis depending on the type of alcohol consumed was also studied. A statistically significant correlation was observed only between beer consumption and the risk of dermatosis occurrence. The authors hypothesised that this could be related to the gluten content in beer and frequent occurrence of disorders of tolerance of this protein fraction among persons suffering from psoriasis [94].

The mechanism of the negative effect of alcohol on the course of psoriasis is not fully understood. This effect may be the result of oxidative stress. Another hypothesis suggests the impact of histamine overproduction, as well as vasodilation, and consequently an increase in the migration of inflammatory cells. Furthermore, alcohol causes increased susceptibility to skin infections, e.g., streptococci, which can trigger the emergence of psoriasis skin symptoms [14,95].

It should also be noted that alcohol consumption is often accompanied by meals with a high saturated fatty acid content and a small intake of vegetables and fruit. Such a diet may contribute to exacerbation of the disease symptoms [6,14].

It has been shown that psoriasis in persons suffering from alcoholism is usually refractory, and the number of adverse effects increases [4,34].

## 14. Conclusions

Unfortunately, no specific nutritional therapy regimens for psoriasis have been established yet. However, numerous studies confirm the positive effect of consumption or elimination of the nutrients and food products mentioned above. When planning the diet of patients with psoriasis, one should also consider co-morbidities and implement actions to prevent the diseases to which these persons are vulnerable [29].

The diet for patients with psoriasis should be varied and tailored to each individual patient. Patients should avoid alcohol, animal fats, red meat, simple sugars, and highly processed food. Large amounts of vegetables and fruits, which are a source of antioxidants, as well as vegetable oils, nuts, and marine fish, supplying fatty acids from the omega-3 family, should be consumed. They should choose whole-grain cereal products and increase the consumption of legumes. In some cases, patients should consider a gluten-free diet and vitamin D supplementation [14,29,34].

A diet that is properly selected and consistently followed by the patient can have a positive impact not only on the course of psoriasis and the prognosis but also on co-morbidities [3,14,29].

Table 2 presents a set of key dietary recommendations for patients with psoriasis.

## 15. Limitations of This Manuscript

The limitations of this work are the lack of a structured review of scientific publications. The authors did not apply the PRISMA guidelines. Some of the referenced studies were older than five years and some articles were not from international journals. In addition, some of the studies cited were conducted only in vitro and require more detailed studies in humans.

## Figures and Tables

**Table 1:** Sources of selected antioxidants [14,48,49,50].

Vitamin A	Vitamin C	Vitamin E	Carotenoids	Flavonoids	Selenium
fish fat, liver, cheese, eggs, butter, Vitamin A is also made from provitamins—carotenoids	raw vegetables and fruits, e.g.,: peppers, parsley leaves, brussels sprouts, broccoli, rosehips, currants, strawberries, citruses	vegetable oils (rapeseed oil, soybean oil, corn oil), nuts, sunflower seeds, wheat germ	vegetables with orange, yellow and green colour: kale, broccoli, brussels sprouts, lettuce, cauliflower, spinach, carrots, red pepper, tomato, white and red cabbage	tomatoes, peppers, onions, broccoli, citrus fruits, apples, grapes, blackcurrants, some cereals (wheat, oats), legumes, red wine, green tea, coffee, cocoa	meat, fish, whole grains (e.g., oats, brown rice), dairy products, brassica vegetables, garlic, onion, asparagus, legumes, nuts (mainly Brazil nuts), sunflower seeds, mushrooms

**Table 2:** Summary of dietary recommendations for patients with psoriasis.

Dietary Aspect	Recommendations
Energy	The energy value of the diet should be individually adapted to the patient. In overweight and obese people, calorie reductions should be implemented to reduce body weight.
Fatty-acids	Limit the supply of saturated fatty acids (found in fatty meats and animal fats such as lard, butter, cream) and trans fats (found in stick margarines, highly processed foods, confectionery products). Increase the supply of omega-3 polyunsaturated fatty acids (especially DHA and EPA), of which marine fish are the best source. The ratio of omega-3 to omega-6 fatty acids should be 1:1.80–1:5. Supplementation with omega-3 fatty acids may be considered.
Carbohydrates	Patients should choose carbohydrate products with a low glycaemic index or glycaemic load. It is recommended to limit the intake of sugar, sweets, fruit preserves, and sweet drinks. Instead of white bread, plain pasta, and white rice, patients should choose whole grain cereals.
Antioxidants	Increase the intake of products that are sources of natural antioxidants (mainly raw fruit and vegetables).
Vitamin D	In case of vitamin D deficiency, supplementation is recommended.
Alcohol	Consumption of alcohol is contraindicated.
Alternative diets	The following alternative diets may be considered: Gluten-free diet Vegetarian diet Mediterranean diet Ketogenic diet
**The diet for patients with psoriasis should be tailored to the individual needs of the patient, their comorbidities, and the treatment they are receiving.**	

## References

[1] Tupikowska M., Zdrojowy-Welna A., Maj J. (2014). Psoriasis as metabolic and cardiovascular risk factor. Pol. Merkur Lek..

[2] WHO Global Report on Psoriasis. https://apps.who.int/iris/handle/10665/204417.

[3] Zuccotti E., Oliveri M., Girometta C., Ratto D., Di Iorio C., Occhinegro A., Rossi P. (2018). Nutritional strategies for psoriasis: Current scientific evidence in clinical trials. Eur. Rev. Med. Pharmacol. Sci..

[4] Placek W., Mieszczak-Woszczyna D. (2011). Dieta w schorzeniach dermatologicznych (II). Znaczenie kwasów omega-3 w leczeniu łuszczycy. Dermatol. Estet..

[5] Szczerkowska-Dobosz A., Komorowska O. (2014). Łuszczyca i miażdżyca—Związek nieprzypadkowy. Dermatol. Dypl..

[6] Trojacka E., Zaleska M., Galus R. (2015). Influence of exogenous and endogenous factors on the course of psoriasis. Pol. Merkur Lek..

[7] Holmannova D., Borska L., Andrys C., Borsky P., Kremlacek J., Hamakova K., Rehacek V., Malkova A., Svadlakova T., Palicka V. (2020). The Impact of Psoriasis and Metabolic Syndrome on the Systemic Inflammation and Oxidative Damage to Nucleic Acids. J. Immunol. Res..

[8] Polic M.V., Miskulin M., Smolic M., Kralik K., Miskulin I., Berkovic M.C., Curcic I.B. (2018). Psoriasis Severity-A Risk Factor of Insulin Resistance Independent of Metabolic Syndrome. Int. J. Environ. Res. Public. Health.

[9] Kanda N., Hoashi T., Saeki H. (2020). Nutrition and Psoriasis. Int. J. Mol. Sci..

[10] Ni C., Chiu M.W. (2014). Psoriasis and comorbidities: Links and risks. Clin. Cosmet. Investig. Dermatol..

[11] Baran A., Kiluk P., Mysliwiec H., Flisiak I. (2017). The role of lipids in psoriasis. Prz. Dermatol..

[12] Gupta S., Syrimi Z., Hughes D.M., Zhao S.S. (2021). Comorbidities in psoriatic arthritis: A systematic review and meta-analysis. Rheumatol. Int..

[13] Choudhary S., Pradhan D., Pandey A., Khan M.K., Lall R., Ramesh V., Puri P., Jain A.K., Thomas G. (2020). The Association of Metabolic Syndrome and Psoriasis: A Systematic Review and Meta-Analysis of Observational Study. Endocr. Metab. Immune Disord. Drug Targets.

[14] Antosik K., Krzęcio-Nieczyporuk E., Kurowska-Socha B. (2017). Diet and nutrition in psoriasis treatment. Hyg. Pub. Health.

[15] Gisondi P., Fostini A., Fossa I., Girolomoni G., Targher G. (2018). Psoriasis and the metabolic syndrome. Clin. Dermatol..

[16] Langan S.M., Seminara N.M., Shin D.B., Troxel A.B., Kimmel S.E., Mehta N.N., Margolis D.J., Gelfand J.M. (2012). Prevalence of metabolic syndrome in patients with psoriasis: A population-based study in the United Kingdom. J. Investig. Dermatol..

[17] Yamazaki F. (2021). Psoriasis: Comorbidities. J. Dermatol..

[18] Atawia R.T., Bunch K.L., Toque H.A., Caldwell R.B., Caldwell R.W. (2019). Mechanisms of obesity-induced metabolic and vascular dysfunctions. Front. Biosci..

[19] Armstrong A.W., Harskamp C.T., Armstrong E.J. (2012). The association between psoriasis and obesity: A systematic review and meta-analysis of observational studies. Nutr. Diabetes.

[20] Snekvik I., Smith C.H., Nilsen T.I.L., Langan S.M., Modalsli E.H., Romundstad P.R., Saunes M. (2017). Obesity, Waist Circumference, Weight Change, and Risk of Incident Psoriasis: Prospective Data from the HUNT Study. J. Investig. Dermatol..

[21] Galluzzo M., Talamonti M., Perino F., Servoli S., Giordano D., Chimenti S., De Simone C., Peris K. (2017). Bioelectrical impedance analysis to define an excess of body fat: Evaluation in patients with psoriasis. J. Dermatol. Treat..

[22] Diniz M.S., Bavoso N.C., Kakehasi A.M., Lauria M.W., Soares M.M., Pinto J.M. (2016). Assessment of adiposity in psoriatic patients by dual energy X-ray absorptiometry compared to conventional methods. An. Bras. Dermatol..

[23] Blake T., Gullick N.J., Hutchinson C.E., Barber T.M. (2020). Psoriatic disease and body composition: A systematic review and narrative synthesis. PLoS ONE.

[24] Barrea L., Macchia P.E., Di Somma C., Napolitano M., Balato A., Falco A., Savanelli M.C., Balato N., Colao A., Savastano S. (2016). Bioelectrical phase angle and psoriasis: A novel association with psoriasis severity, quality of life and metabolic syndrome. J. Transl. Med..

[25] Budu-Aggrey A., Brumpton B., Tyrrell J., Watkins S., Modalsli E.H., Celis-Morales C., Ferguson L.D., Vie G., Palmer T., Fritsche L.G. (2019). Evidence of a causal relationship between body mass index and psoriasis: A mendelian randomization study. PLoS Med..

[26] Sahi F.M., Masood A., Danawar N.A., Mekaiel A., Malik B.H. (2020). Association between Psoriasis and Depression: A Traditional Review. Cureus.

[27] Bremner J.D., Moazzami K., Wittbrodt M.T., Nye J.A., Lima B.B., Gillespie C.F., Rapaport M.H., Pearce B.D., Shah A.J., Vaccarino V. (2020). Diet, Stress and Mental Health. Nutrients.

[28] Balbás G.M., Regaña M.S., Millet P.U. (2011). Study on the use of omega-3 fatty acids as a therapeutic supplement in treatment of psoriasis. Clin. Cosmet. Investig. Dermatol..

[29] Barrea L., Nappi F., Di Somma C., Savanelli M.C., Falco A., Balato A., Balato N., Savastano S. (2016). Environmental Risk Factors in Psoriasis: The Point of View of the Nutritionist. Int. J. Environ. Res. Public Health.

[30] Gołąbek K., Regulska-Ilow B. (2017). Dietary support of pharmacological psoriasis treatment. Hyg. Pub. Health.

[31] Jensen P., Zachariae C., Christensen R., Geiker N.R., Schaadt B.K., Stender S., Hansen P.R., Astrup A., Skov L. (2013). Effect of weight loss on the severity of psoriasis: A randomized clinical study. JAMA Dermatol..

[32] Jensen P., Christensen R., Zachariae C., Geiker N.R., Schaadt B.K., Stender S., Hansen P.R., Astrup A., Skov L. (2016). Long-term effects of weight reduction on the severity of psoriasis in a cohort derived from a randomized trial: A prospective observational follow-up study. Am. J. Clin. Nutr..

[33] Gisondi P., Del Giglio M., Di Francesco V., Zamboni M., Girolomoni G. (2008). Weight loss improves the response of obese patients with moderate-to-severe chronic plaque psoriasis to low-dose cyclosporine therapy: A randomized, controlled, investigator-blinded clinical trial. Am. J. Clin. Nutr..

[34] Wasiluk D., Ostrowska L., Stefańska E. (2012). Can an adequate diet be helpful in the treatment of psoriasis vulgaris?. Med. Og. Nauki. Zdr..

[35] Sicinska P., Pytel E., Kurowska J., Koter-Michalak M. (2015). Supplementation with omega fatty acids in various diseases. Postepy Hig. I Med. Dosw..

[36] Millsop J.W., Bhatia B.K., Debbaneh M., Koo J., Liao W. (2014). Diet and psoriasis, part III: Role of nutritional supplements. J. Am. Acad. Dermatol..

[37] Adil M., Singh P., Maheshwari K. (2017). Clinical evaluation of omega-3 fatty acids in psoriasis. Prz. Dermatol..

[38] Owczarczyk-Saczonek A., Purzycka-Bohdan D., Nedoszytko B., Reich A., Szczerkowska-Dobosz A., Bartosinska J., Batycka-Baran A., Czajkowski R., Dobrucki I., Dobrucki L. (2020). Pathogenesis of psoriasis in the “omic” era. Part III. Metabolic disorders, metabolomics, nutrigenomics in psoriasis. Postepy Dermatol. I Alergol..

[39] Ashcroft F.J., Mahammad N., Midtun Flatekvål H., Jullumstrø Feuerherm A., Johansen B. (2020). cPLA_2_α Enzyme Inhibition Attenuates Inflammation and Keratinocyte Proliferation. Biomolecules.

[40] Shao S., Chen J., Swindell W.R., Tsoi L.C., Xing X., Ma F., Uppala R., Sarkar M.K., Plazyo O., Billi A.C. (2021). Phospholipase A2 enzymes represent a shared pathogenic pathway in psoriasis and pityriasis rubra pilaris. JCI Insight.

[41] Barrea L., Macchia P.E., Tarantino G., Di Somma C., Pane E., Balato N., Napolitano M., Colao A., Savastano S. (2015). Nutrition: A key environmental dietary factor in clinical severity and cardio-metabolic risk in psoriatic male patients evaluated by 7-day food-frequency questionnaire. J. Transl. Med..

[42] Chen X., Hong S., Sun X., Xu W., Li H., Ma T., Zheng Q., Zhao H., Zhou Y., Qiang Y. (2020). Efficacy of fish oil and its components in the management of psoriasis: A systematic review of 18 randomized controlled trials. Nutr. Rev..

[43] Mendivil C.O. (2020). Dietary Fish, Fish Nutrients, and Immune Function: A Review. Front. Nutr..

[44] Ingkapairoj K., Chularojanamontri L., Chaiyabutr C., Silpa-Archa N., Wongpraparut C., Bunyaratavej S. (2021). Dietary habits and perceptions of psoriatic patients: Mediterranean versus Asian diets. J. Dermatol. Treat..

[45] Yang S.J., Chi C.C. (2019). Effects of fish oil supplement on psoriasis: A meta-analysis of randomized controlled trials. BMC Complement. Altern. Med..

[46] Barrea L., Balato N., Di Somma C., Macchia P.E., Napolitano M., Savanelli M.C., Esposito K., Colao A., Savastano S. (2015). Nutrition and psoriasis: Is there any association between the severity of the disease and adherence to the Mediterranean diet?. J. Transl. Med..

[47] Winiarska-Mieczan A., Mieczan T., Wójcik G. (2020). Importance of Redox Equilibrium in the Pathogenesis of Psoriasis-Impact of Antioxidant-Rich Diet. Nutrients.

[48] Ratajczak M., Gietka-Czernel M. (2016). The influence of selenium to human health. Post N Med..

[49] Janda K., Kasprzak M., Wolska J. (2015). Vitamin C—Structure, properties, occurrence and functions. Pomeranian J. Life Sci..

[50] Zalega J., Szostak-Węgierek D. (2013). Nutrition in cancer prevention. Part II. Minerals, vitamins, polyunsaturated fatty acids, probiotics, prebiotics. Probl. Hig. Epidemiol..

[51] Halamek D. (2016). Anti-aging properties of vitamin D. Acad. Aesthet. Anti-Aging Med..

[52] Wu Q., Xu Z., Dan Y.L., Zhao C.N., Mao Y.M., Liu L.N., Pan H.F. (2020). Seasonality and global public interest in psoriasis: An infodemiology study. Postgrad. Med. J..

[53] Finamor D.C., Sinigaglia-Coimbra R., Neves L.C., Gutierrez M., Silva J.J., Torres L.D., Surano F., Neto D.J., Novo N.F., Juliano Y. (2013). A pilot study assessing the effect of prolonged administration of high daily doses of vitamin D on the clinical course of vitiligo and psoriasis. Dermato-Endocrinol..

[54] Gaál J., Lakos G., Szodoray P., Kiss J., Horváth I., Horkay E., Nagy G., Szegedi A. (2009). Immunological and clinical effects of alphacalcidol in patients with psoriatic arthropathy: Results of an open, follow-up pilot study. Acta. Dermatol. Venereol..

[55] Tajjour R., Baddour R., Redwan F., Hassan F. (2018). The relationship between psoriasis and serum levels of vitamin D. JAMMR.

[56] Faraji S., Alizadeh M. (2020). Mechanistic Effects of Vitamin D Supplementation on Metabolic Syndrome Components in Patients with or without Vitamin D Deficiency. J. Obes. Metab. Syndr..

[57] Barrea L., Savanelli M.C., Di Somma C., Napolitano M., Megna M., Colao A., Savastano S. (2017). Vitamin D and its role in psoriasis: An overview of the dermatologist and nutritionist. Rev. Endocr. Metab. Disord..

[58] de la Guía-Galipienso F., Martínez-Ferran M., Vallecillo N., Lavie C.J., Sanchis-Gomar F., Pareja-Galeano H. (2021). Vitamin D and cardiovascular health. Clin. Nutr..

[59] EFSA Panel on Dietetic Products, Nutrition and Allergies (NDA) (2012). Scientific Opinion on the Tolerable Upper Intake Level of vitamin D. EFSA J..

[60] Takahashi M., Takahashi K., Abe S., Yamada K., Suzuki M., Masahisa M., Endo M., Abe K., Inoue R., Hoshi H. (2020). Improvement of Psoriasis by Alteration of the Gut Environment by Oral Administration of Fucoidan from. Mar. Drugs.

[61] Codoñer F.M., Ramírez-Bosca A., Climent E., Carrión-Gutierrez M., Guerrero M., Pérez-Orquín J.M., Horga de la Parte J., Genovés S., Ramón D., Navarro-López V. (2018). Gut microbial composition in patients with psoriasis. Sci. Rep..

[62] Eppinga H., Sperna Weiland C.J., Thio H.B., van der Woude C.J., Nijsten T.E., Peppelenbosch M.P., Konstantinov S.R. (2016). Similar Depletion of Protective Faecalibacterium prausnitzii in Psoriasis and Inflammatory Bowel Disease, but not in Hidradenitis Suppurativa. J. Crohn’s Colitis.

[63] Huang L., Gao R., Yu N., Zhu Y., Ding Y., Qin H. (2019). Dysbiosis of gut microbiota was closely associated with psoriasis. Sci. China Life Sci..

[64] Scher J.U., Ubeda C., Artacho A., Attur M., Isaac S., Reddy S.M., Marmon S., Neimann A., Brusca S., Patel T. (2015). Decreased bacterial diversity characterizes the altered gut microbiota in patients with psoriatic arthritis, resembling dysbiosis in inflammatory bowel disease. Arthritis Rheumatol..

[65] Tan L., Zhao S., Zhu W., Wu L., Li J., Shen M., Lei L., Chen X., Peng C. (2018). The Akkermansia muciniphila is a gut microbiota signature in psoriasis. Exp. Dermatol..

[66] Koper M., Wozniacka A., Robak E. (2020). The intestinal microbiota in psoriasis. Postepy Hig. I Med. Dosw..

[67] Szabo-Fodor J., Bonai A., Bota B., Egyed L., Lakatos F., Papai G., Zsolnai A., Glavits R., Horvatovich K., Kovacs M. (2017). Physiological Effects of Whey- and Milk-Based Probiotic Yogurt in Rats. Pol. J. Microbiol..

[68] Kariyawasam K.M.G.M., Lee N.K., Paik H.D. (2021). Fermente.ed dairy products as delivery vehicles of novel probiotic strains isolated from traditional fermented Asian foods. J. Food Sci. Technol..

[69] Navarro-López V., Núñez-Delegido E., Ruzafa-Costas B., Sánchez-Pellicer P., Agüera-Santos J., Navarro-Moratalla L. (2021). Probiotics in the Therapeutic Arsenal of Dermatologists. Microorganisms.

[70] Navarro-López V., Martínez-Andrés A., Ramírez-Boscá A., Ruzafa-Costas B., Núñez-Delegido E., Carrión-Gutiérrez M.A., Prieto-Merino D., Codoñer-Cortés F., Ramón-Vidal D., Genovés-Martínez S. (2019). Efficacy and Safety of Oral Administration of a Mixture of Probiotic Strains in Patients with Psoriasis: A Randomized Controlled Clinical Trial. Acta Dermatol. Venereol..

[71] Li Y., Zheng Y., Zhang Y., Yang Y., Wang P., Imre B., Wong A.C.Y., Hsieh Y.S.Y., Wang D. (2021). Brown Algae Carbohydrates: Structures, Pharmaceutical Properties, and Research Challenges. Mar. Drugs.

[72] Shen S., Chen X., Shen Z., Chen H. (2021). Marine Polysaccharides for Wound Dressings Application: An Overview. Pharmaceutics.

[73] Conde T.A., Neves B.F., Couto D., Melo T., Neves B., Costa M., Silva J., Domingues P., Domingues M.R. (2021). Microalgae as Sustainable Bio-Factories of Healthy Lipids: Evaluating Fatty Acid Content and Antioxidant Activity. Mar. Drugs.

[74] Rocha C.P., Pacheco D., Cotas J., Marques J.C., Pereira L., Gonçalves A.M.M. (2021). Seaweeds as Valuable Sources of Essential Fatty Acids for Human Nutrition. Int. J. Environ. Res. Public Health.

[75] Dalheim L., Svenning J.B., Olsen R.L. (2021). In vitro intestinal digestion of lipids from the marine diatom Porosira glacialis compared to commercial LC n-3 PUFA products. PLoS ONE.

[76] Verspreet J., Soetemans L., Gargan C., Hayes M., Bastiaens L. (2021). Nutritional Profiling and Preliminary Bioactivity Screening of Five Micro-Algae Strains Cultivated in Northwest Europe. Foods.

[77] Hughes L.J., Black L.J., Sherriff J.L., Dunlop E., Strobel N., Lucas R.M., Bornman J.F. (2018). Vitamin D Content of Australian Native Food Plants and Australian-Grown Edible Seaweed. Nutrients.

[78] Göring H. (2018). Vitamin D in Nature: A Product of Synthesis and/or Degradation of Cell Membrane Components. Biochemistry.

[79] Grether-Beck S., Marini A., Jaenicke T., Brenden H., Felsner I., Aue N., Brynjolfsdottir A., Krutmann J. (2021). Blue Lagoon Algae Improve Uneven Skin Pigmentation: Results from in vitro Studies and from a Monocentric, Randomized, Double-Blind, Vehicle-Controlled, Split-Face Study. Ski. Pharm. Physiol..

[80] Barrea L., Muscogiuri G., Di Somma C., Annunziata G., Megna M., Falco A., Balato A., Colao A., Savastano S. (2018). Coffee consumption, metabolic syndrome and clinical severity of psoriasis: Good or bad stuff?. Arch. Toxicol..

[81] Baspinar B., Eskici G., Ozcelik A.O. (2017). How coffee affects metabolic syndrome and its components. Food Funct..

[82] Gökcen B.B., Şanlier N. (2019). Coffee consumption and disease correlations. Crit. Rev. Food Sci. Nutr..

[83] Grosso G., Godos J., Galvano F., Giovannucci E.L. (2017). Coffee, Caffeine, and Health Outcomes: An Umbrella Review. Annu. Rev. Nutr..

[84] Madeira M.H., Boia R., Ambrósio A.F., Santiago A.R. (2017). Having a Coffee Break: The Impact of Caffeine Consumption on Microglia-Mediated Inflammation in Neurodegenerative Diseases. Mediat. Inflamm..

[85] Sharif K., Watad A., Bragazzi N.L., Adawi M., Amital H., Shoenfeld Y. (2017). Coffee and autoimmunity: More than a mere hot beverage!. Autoimmun Rev..

[86] Hall S., Desbrow B., Anoopkumar-Dukie S., Davey A.K., Arora D., McDermott C., Schubert M.M., Perkins A.V., Kiefel M.J., Grant G.D. (2015). A review of the bioactivity of coffee, caffeine and key coffee constituents on inflammatory responses linked to depression. Food Res. Int..

[87] Zampelas A., Panagiotakos D.B., Pitsavos C., Chrysohoou C., Stefanadis C. (2004). Associations between coffee consumption and inflammatory markers in healthy persons: The ATTICA study. Am. J. Clin. Nutr..

[88] Li W., Han J., Qureshi A.A. (2012). No association between coffee and caffeine intake and risk of psoriasis in US women. Arch. Dermatol..

[89] Favari C., Righetti L., Tassotti M., Gethings L.A., Martini D., Rosi A., Antonini M., Rubert J., Manach C., Dei Cas A. (2021). Metabolomic Changes after Coffee Consumption: New Paths on the Block. Mol. Nutr. Food Res..

[90] Passali M., Josefsen K., Frederiksen J.L., Antvorskov J.C. (2020). Current Evidence on the Efficacy of Gluten-Free Diets in Multiple Sclerosis, Psoriasis, Type 1 Diabetes and Autoimmune Thyroid Diseases. Nutrients.

[91] Ungprasert P., Wijarnpreecha K., Kittanamongkolchai W. (2017). Psoriasis and Risk of Celiac Disease: A Systematic Review and Meta-analysis. Indian J. Dermatol..

[92] Bhatia B.K., Millsop J.W., Debbaneh M., Koo J., Linos E., Liao W. (2014). Diet and psoriasis, part II: Celiac disease and role of a gluten-free diet. J. Am. Acad. Dermatol..

[93] Dhattarwal N., Mahajan V.K., Mehta K.S., Chauhan P.S., Yadav R.S., Sharma S.B., Sharma A., Sharma R., Rana A., Sondhi M. (2020). The association of anti-gliadin and anti-transglutaminase antibodies and chronic plaque psoriasis in Indian patients: Preliminary results of a descriptive cross-sectional study. Australas. J. Dermatol..

[94] Qureshi A.A., Dominguez P.L., Choi H.K., Han J., Curhan G. (2010). Alcohol intake and risk of incident psoriasis in US women: A prospective study. Arch. Dermatol..

[95] Gelfand J.M., Dommasch E.D., Shin D.B., Azfar R.S., Kurd S.K., Wang X., Troxel A.B. (2009). The risk of stroke in patients with psoriasis. J. Investig. Dermatol..

[96] Korovesi A., Dalamaga M., Kotopouli M., Papadavid E. (2019). Adherence to the Mediterranean diet is independently associated with psoriasis risk, severity, and quality of life: A cross-sectional observational study. Int. J. Dermatol..

[97] Phan C., Touvier M., Kesse-Guyot E., Adjibade M., Hercberg S., Wolkenstein P., Chosidow O., Ezzedine K., Sbidian E. (2018). Association Between Mediterranean Anti-inflammatory Dietary Profile and Severity of Psoriasis: Results From the NutriNet-Santé Cohort. JAMA Dermatol..

[98] Molina-Leyva A., Cuenca-Barrales C., Vega-Castillo J.J., Ruiz-Carrascosa J.C., Ruiz-Villaverde R. (2019). Adherence to Mediterranean diet in Spanish patients with psoriasis: Cardiovascular benefits?. Dermatol. Ther..

[99] Caso F., Navarini L., Carubbi F., Picchianti-Diamanti A., Chimenti M.S., Tasso M., Currado D., Ruscitti P., Ciccozzi M., Annarumma A. (2020). Mediterranean diet and Psoriatic Arthritis activity: A multicenter cross-sectional study. Rheumatol. Int..

[100] Herbert D., Franz S., Popkova Y., Anderegg U., Schiller J., Schwede K., Lorz A., Simon J.C., Saalbach A. (2018). High-Fat Diet Exacerbates Early Psoriatic Skin Inflammation Independent of Obesity: Saturated Fatty Acids as Key Players. J. Investig. Dermatol..

[101] Nakamizo S., Honda T., Adachi A., Nagatake T., Kunisawa J., Kitoh A., Otsuka A., Dainichi T., Nomura T., Ginhoux F. (2017). High fat diet exacerbates murine psoriatic dermatitis by increasing the number of IL-17-producing γδ T cells. Sci. Rep..

[102] Locker F., Leitner J., Aminzadeh-Gohari S., Weber D.D., Sanio P., Koller A., Feichtinger R.G., Weiss R., Kofler B., Lang R. (2020). The Influence of Ketogenic Diets on Psoriasiform-Like Skin Inflammation. J. Investig. Dermatol..

[103] Barrea L., Megna M., Cacciapuoti S., Frias-Toral E., Fabbrocini G., Savastano S., Colao A., Muscogiuri G. (2020). Very low-calorie ketogenic diet (VLCKD) in patients with psoriasis and obesity: An update for dermatologists and nutritionists. Crit. Rev. Food Sci. Nutr..

[104] Muscogiuri G., Barrea L., Laudisio D., Pugliese G., Salzano C., Savastano S., Colao A. (2019). The management of very low-calorie ketogenic diet in obesity outpatient clinic: A practical guide. J. Transl. Med..

[105] Castaldo G., Pagano I., Grimaldi M., Marino C., Molettieri P., Santoro A., Stillitano I., Romano R., Montoro P., D’Ursi A.M. (2021). Effect of Very-Low-Calorie Ketogenic Diet on Psoriasis Patients: A Nuclear Magnetic Resonance-Based Metabolomic Study. J. Proteome Res..

[106] Castaldo G., Rastrelli L., Galdo G., Molettieri P., Rotondi Aufiero F., Cereda E. (2020). Aggressive weight-loss program with a ketogenic induction phase for the treatment of chronic plaque psoriasis: A proof-of-concept, single-arm, open-label clinical trial. Nutrition.

[107] Zychowska M., Batycka-Baran A., Bieniek A., Baran W. (2014). Folate supplementation in patients with psoriasis treated with methotrexate—Effect on safety and efficacy. Prz. Dermatol..

